# Effects of DNA topoisomerase IIα splice variants on acquired drug resistance

**DOI:** 10.20517/cdr.2019.117

**Published:** 2020-02-27

**Authors:** Terry S. Elton, Hatice Gulcin Ozer, Jack C. Yalowich

**Affiliations:** 1 Division of Pharmaceutics and Pharmacology, College of Pharmacy, The Ohio State University, Columbus, OH 43210, USA.; 2 Department of Biomedical Informatics, College of Medicine, The Ohio State University, Columbus, OH 43210, USA.

**Keywords:** DNA topoisomerase IIα, chemoresistance, alternative splicing, intron retention, topoisomerase IIα interfacial inhibitors/poisons

## Abstract

DNA topoisomerase IIα (170 kDa, TOP2α/170) induces transient DNA double-strand breaks in proliferating cells to resolve DNA topological entanglements during chromosome condensation, replication, and segregation. Therefore, TOP2α/170 is a prominent target for anticancer drugs whose clinical efficacy is often compromised due to chemoresistance. Although many resistance mechanisms have been defined, acquired resistance of human cancer cell lines to TOP2α interfacial inhibitors/poisons is frequently associated with a reduction of Top2α/170 expression levels. Recent studies by our laboratory, in conjunction with earlier findings by other investigators, support the hypothesis that a major mechanism of acquired resistance to TOP2α-targeted drugs is due to alternative RNA processing/splicing. Specifically, several TOP2α mRNA splice variants have been reported which retain introns and are translated into truncated TOP2α isoforms lacking nuclear localization sequences and subsequent dysregulated nuclear-cytoplasmic disposition. In addition, intron retention can lead to truncated isoforms that lack both nuclear localization sequences and the active site tyrosine (Tyr805) necessary for forming enzyme-DNA covalent complexes and inducing DNA damage in the presence of TOP2α-targeted drugs. Ultimately, these truncated TOP2α isoforms result in decreased drug activity against TOP2α in the nucleus and manifest drug resistance. Therefore, the complete characterization of the mechanism(s) regulating the alternative RNA processing of TOP2α pre-mRNA may result in new strategies to circumvent acquired drug resistance. Additionally, novel TOP2α splice variants and truncated TOP2α isoforms may be useful as biomarkers for drug resistance, prognosis, and/or direct future TOP2α-targeted therapies.

## INTRODUCTION

The human DNA topoisomerase IIα (170 kDa, TOP2α/170) enzyme functions as a homodimer with the active site Tyr805 residues in each subunit initiating reversible transesterification reactions to generate TOP2α/170-DNA covalent cleavage complexes^[1–4]^. These transient TOP2α/170 mediated double-strand DNA breaks are essential in proliferating cells so that entanglements which occur during DNA repair, recombination, replication, transcription, and segregation can be resolved by allowing the passage of double-stranded DNA segments through these openings^[1–4]^. Given that TOP2α/170 enzymatic activity is necessary for cell survival, TOP2α interfacial inhibitors/poisons (e.g., etoposide, mitoxantrone, doxorubicin, daunorubicin, and analogs) are widely exploited as anticancer drugs^[5–8]^. These therapeutic agents exert their cytotoxic effects by impeding the reversal of the TOP2α/170-DNA covalent cleavage complexes, which subsequently leads to the accumulation of DNA breaks and ultimately cell death^[5–8]^.

TOP2α poisons are commonly used as chemotherapeutic agents in adults and pediatric patients to treat a wide variety solid tumors, leukemias, and lymphomas^[9–11]^. For example, cisplatin/etoposide is first-line treatment for small cell lung cancer^[12,13]^; doxorubicin and epirubicin are used in combination with other drugs as a preoperative/adjuvant therapy regimen for the treatment of breast cancer^[14,15]^; and daunorubicin and mitoxantrone are used in treating acute myeloid leukemia (AML)^[16,17]^.

Although TOP2α poisons are extensively utilized, the efficacy of these important drugs is often compromised due to acquired chemoresistance^[18–21]^. While many chemoresistant mechanisms have been defined^[22,23]^, acquired resistance to TOP2α poisons is frequently associated with decreased TOP2α/170 expression levels or altered sub-cellular localization of TOP2α/170 given that the cytotoxic activity of these drugs is dependent upon the formation of TOP2α/170-DNA covalent cleavage complexes^[18–21]^. In this review, we focus on the molecular mechanisms underlying the decreased TOP2α/170 expression levels in chemoresistant cell lines due to alternative RNA processing.

## ALTERNATIVE SPLICING

Alternative splicing is a process by which a single pre-mRNA is matured into multiple mRNA isoforms that can contribute to transcriptomic and proteomic diversity^[24]^. RNA-seq data predict that over 95% of human genes generate at least two alternative spliced mRNA isoforms^[24]^. Several modes of alternative splicing of a pre-mRNA have been described: exon skipping, differential inclusion of an exon, alternative splice (5’ splice or 3’ splice) site selection, and intron retention^[24]^. Intron-retaining mRNA transcripts are susceptible to nuclear intron detention^[25]^, or nonsense mediated decay^[26,27]^, and as a consequence gene expression is reduced at the post-transcriptional level. However, some intron-retaining mRNA transcripts leave the nucleus and undergo translation to produce new protein isoforms with novel functions^[28–31]^. Such seems to be the case with a number of documented TOP2α mRNA splice variants, which retain introns, are translated into truncated TOP2α isoforms, and play a role in mediating TOP2α poison chemoresistance in various cell lines^[32–36]^.

## THE HUMAN *TOP2α* GENE AND TOP2α/170 PROTEIN EXPRESSION

**Figure 1.**
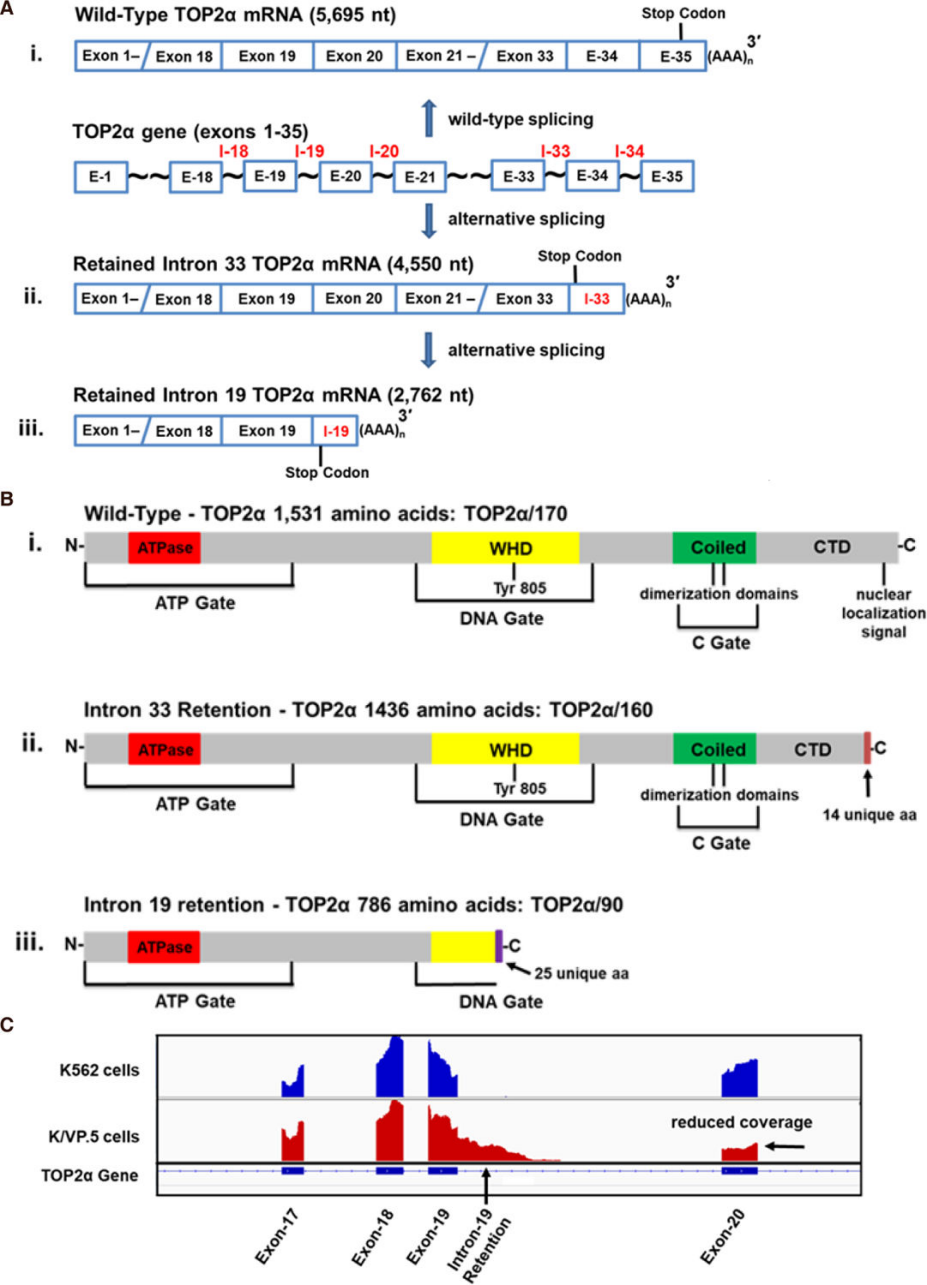
Schematic representation of the human *TOP2α* gene, TOP2α mRNAs, TOP2α protein, and visualization of RNA-seq results. A: the human *TOP2α* gene is comprised of 35 exons. At least three mature mRNA transcripts (i-iii) can be transcribed from the human *TOP2α* gene. Two of these mRNAs harbor retained and processed introns; B: the three TOP2α mRNAs encode three distinct TOP2α protein isoforms. Depicted are the ATP gate, which harbors the ATPase domain; the DNA gate, which includes the winged-helix domain and harbors the active site tyrosine, Tyr805; the C gate, which comprises the coiled-coil region (coiled domain) and the characterized dimerization sequences, DD1053–1069 and DD1121–1143^[41–45]^; and the C-terminal domain, which contains the defined nuclear localization signal NLS1454–1497^[46,47]^; C: visualization of retained intron 19 of TOP2α RNA-seq genome coverage tracks showing the intron 19 retention event in K/VP.5 cells. RNA-seq raw reads from K562 and K/VP.5 RNA samples were mapped to the human reference genome GRCh38 using Hierarchical Indexing for Spliced Alignment for Transcripts v.2.1.0^[48]^ and visualized using the Integrative Genomics Viewer^[49]^. Reduced coverage denoted for Exon 20 indicates fewer full length TOP2α/170 reads in K/VP.5 cells. (A, B) Images adapted in part from Figures 1A, B published originally in the Journal of Pharmacology and Experimental Therapeutics; Kanagasabai *et al.*^[35]^, 2017. TOP2α: topoisomerase IIα; WHD: winged-helix domain; CTD: C-terminal domain; DD: dimerization domains

The human *TOP2α* gene comprises 35 exons, spans ~30 kb (NCBI Reference Sequence: NG_027678.2) [Figure 1A]^[37]^, and has been mapped to chromosome 17q21–22^[38]^. A 5695 nucleotide (nt) mRNA (NCBI Reference Sequence: NM_001067.4) [Figure 1A–i] is matured from the *TOP2α* gene and the open reading frame encodes a protein comprising 1531 amino acids (aa), with a calculated molecular weight of 174,386 Da (i.e., TOP2α/170) [Figure 1B–i]^[37]^. TOP2α exons 1-12 encode the ATP binding domain^[37]^ near the N-terminus and acts as a gate (ATP gate) [Figure 1B–i] when two TOP2α/170 subunits homodimerize^[39,40]^. When the ATP gate is open, one DNA duplex (designated the G- or “gate”-segment) is loaded into the enzyme cavity and a transient double-strand DNA break is generated (i.e., TOP2α/170-DNA covalent cleavage complex)^[39,40]^ within the DNA gate [Figure 1B–i], which is encoded by TOP2α exons 13-27^[37]^. The transesterification reaction, which is mediated by the active site Tyr805 residue on each monomer, is encoded by exon 20^[37]^. Subsequently, the “transfer”-segment (T-segment) is captured within the ATP gate upon ATP binding and is transported through the DNA gate^[39,40]^. This intact DNA duplex then exits from the open C gate^[39,40]^, which comprises the coiled-coil region (coiled domain) and the C-terminal domain from each monomer encoded by TOP2α exons 28-35^[37]^ [Figure 1B–i]. After T-segment strand passage and ATP hydrolysis, the G-segment double-strand DNA break is resealed and free Tyr805 residues present in each TOP2α/170 subunit are regenerated. Finally, the ATP gate is reopened, and this processive enzyme is reset for another round of catalytic activity^[39,40]^. Given the complexity of this enzyme’s reaction cycle, truncated TOP2α isoforms, translated as a result of alternative RNA splicing, may exhibit atypical TOP2α functionality and response to targeted agents.

## TOP2α/160 (INTRON 33 RETENTION) AND CHEMORESISTANCE

Several acquired and innate resistant models have been reported, which involve intron retention due to alternative RNA processing of TOP2α mRNA^[32–36]^. Harker *et al.*^[50]^ generated a mitoxantrone resistant human AML (HL-60) cell line designated HL-60/MX2 (35-fold resistant), by stepwise drug exposure from 1.7 to 170 nM. HL-60/MX2 cells were found to be cross-resistant to a number of TOP2α poisons including etoposide, amsacrine, teniposide, daunorubicin, and doxorubicin^[50]^. Compared to parental HL-60 cells, HL-60/MX2 cells contained reduced TOP2α/170 protein levels and expressed a novel truncated TOP2α isoform migrating at ~160 kDa (TOP2α/160) that localized predominantly to the cytoplasm^[51]^. Interestingly, TOP2α/160 (1436 aa and a calculated molecular weight of 164,052 Da) is the translation product of a TOP2α mRNA (4550 nt) that harbors exons 1–33 and retains a processed intron 33 (125 nt) that contains an in-frame stop codon and a consensus poly(A) site [Figure 1A–ii]^[32]^. As a result of intron 33 retention and processing, TOP2α/160 is missing the C-terminal 108 aa present in TOP2α/170 (1531 aa), which are replaced by 14 unique aa encoded by translation of the exon 33/intron 33 “read-through” [Figure 1B–ii]^[32]^. Importantly, TOP2α/160 is missing the well-characterized nuclear localization signal (NLS) NLS1454–1497^[46,47]^ [Figure 1B–ii]. This isoform is also missing a “chromatin tether” sequence, which interacts with histone tails and anchors TOP2α/170 to nucleosomes^[52]^. These deletions may account for the accumulation of TOP2α/160 in the cytoplasm^[32]^.

Similarly, Feldhoff *et al.*^[53]^ generated a resistant H209 small cell lung cancer cell line, designated H209/V6 (22-fold resistant), by stepwise selection in etoposide (from 0.2 to 6 µM). These investigators demonstrated that, compared to parental H209 cells expressing TOP2α/170, H209/V6 cells only expressed a TOP2α/160 isoform^[53]^. Additionally, it was shown by immunocytochemistry and cytoplasm/nuclear fractionation studies that TOP2α/160 was primarily localized in the cytoplasm^[54]^. Yu *et al.*^[33]^ subsequently characterized a TOP2α mRNA splice variant (7090 nt) expressed in the etoposide resistant H209/V6 cell line that harbored exons 1–33, the entire intron 33, and included exons 34 and 35 [see Figure 1A for orientation]. Although this mRNA is much longer than the 4550 nt transcript from HL60/MX2 cells^[32]^, it is still translated into the same TOP2α/160 (1436 aa, 164,052 Da) isoform described above by Harker *et al.*^[32]^ [Figure 1A–ii] due to the in-frame stop codon present in retained intron 33, loss of the canonical NLS, and consequent aberrant localization in the cytoplasm^[33,53,54]^.

Mo and Beck^[34]^ characterized TOP2α mRNA splice variants in TOP2α poison sensitive T-lineage tumor cell lines (e.g., CEM, Jurkat, and H9). One of four TOP2α mRNA splice variants characterized in CEM cells was identical to the transcript that was described above by Harker *et al*.^[32]^ with exons 1–33, followed by a retained and processed intron 33 (4550 nt), and again encoded the identical TOP2α/160 (1436 aa, 164,052 Da). This truncated TOP2α isoform and others generated from intron retention in T-cell lines were lacking the canonical NLS and all were detected in cytoplasmic extracts^[34]^. Interestingly, normal T-cells contained only TOP2α/170, prompting these investigators to suggest that splice variants of TOP2α play a role in leukemogenesis, although no further investigations to explore this possibility have been reported.

Together, these previous reports suggest that intron retention can play a role in generation of truncated TOP2α isoforms secondary to alternative RNA processing. The production of truncated TOP2α isoforms can be determinants of drug resistance and/or play a role tumor cell biology not yet characterized.

## TOP2α/90 (INTRON 19 RETENTION) AND CHEMORESISTANCE

Our laboratory has also investigated the molecular mechanisms which lead to decreased TOP2α/170 expression levels in acquired chemoresistance. Resistant human leukemia K562 cells were generated by intermittent then continuous treatment with 0.5 µM etoposide followed by limiting dilution to isolate and then characterize a clonal K/VP.5 cell line^[55]^. Compared to parental K562 cells, the K/VP.5 subline was 30-fold resistant to etoposide and cross-resistant to teniposide, mitoxantrone, doxorubicin, and amsacrine^[56]^. This multi-drug resistance was not mediated by overexpression of ABCB1^[56]^. K/VP.5 cells exhibited reduced TOP2α/170 mRNA (by Northern blot analysis) with no change in transcription compared to K562 cells^[55]^. In addition, using an antibody generated from the C-terminal 70 kDa of TOP2α, immunoassays of cells lysates demonstrated reduced TOP2α/170 protein levels in K/VP.5 compared to K562 cells^[55,56]^.

**Figure 2.**
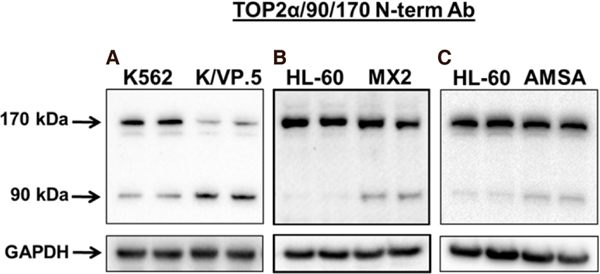
A novel human TOP2α/90 isoform is overexpressed in acquired resistance to TOP2α-targeted drugs etoposide, mitoxantrone, and amsacrine. A: TOP2α immunoassay utilizing K562 and K/VP.5^[35,36,55,56]^ cell lysates; B: TOP2α immunoassay utilizing HL-60 and HL-60/MX2^[32,50,51]^ cell lysates; C: TOP2α immunoassay utilizing HL-60 and HL-60/AMSA^[57]^ cell lysates. The immunoblots were probed with an antibody specific for the N-terminal portion of TOP2α/170 (i.e., amino acids 14–27, denoted N-terminal Ab). (A) Image is from Figure 2A published originally in the *Journal of Pharmacology and Experimental Therapeutics*; Kanagasabai *et al.*^[35]^, 2017. TOP2α: topoisomerase IIα

Surprisingly, additional immunoblotting experiments using a N-terminal specific TOP2α/170 antibody (generated against amino acids 14-27) revealed the presence of two major TOP2α proteins, the expected wild-type TOP2α/170 isoform and a novel 90 kDa isoform, TOP2α/90 [Figure 2A]^[35,36]^. Compared to parental K562 cells, the expression level of TOP2α/170 was attenuated as expected but TOP2α/90 was increased in K/VP.5 cells [Figure 2A]^[35,36]^. Immunoassays utilizing cell lysates from two additional TOP2α-poison resistant cell lines, HL-60/MX2 (mitoxantrone-resistant)^[32]^ and HL-60/AMSA (amsacrine-resistant)^[57]^, also demonstrated greater TOP2α/90 protein levels compared to parental HL-60 cells [Figure 2B and C].

Using 3’-rapid amplification of cDNA ends (3’-RACE), followed by PCR and sequencing, analyses revealed that TOP2α/90 mRNA (2762 nt) shares the first 19 exons with the TOP2α/170 transcript. However, the TOP2α/90 mRNA retains a processed intron 19 (380 nt) that harbors an in-frame stop codon, and two consensus poly(A) sites [Figure 1A–iii]^[35]^. TOP2α/90 mRNA lacks the published TOP2α/170 transcript sequences from exon 20 to 35, and harbors a novel 3’-untranslated region (302 nt) [Figure 1A–iii]^[35]^. TOP2α/90 mRNA intron 19 retention was validated by mapping RNA-seq raw reads [Figure 1C].

The TOP2α/90 mRNA encodes a truncated TOP2α protein isoform of 786 aa with a calculated molecular weight of 90,076 Da, which is approximately one half the size of the wild-type TOP2α/170 protein (i.e., 1531 aa, 174,385 Da) [Figure 1B]^[35,36]^. Although TOP2α/90 is identical to TOP2α/170 for the first 761 aa, this protein is missing the C-terminal 770 aa present in TOP2α/170, which are replaced with 25 unique amino acids encoded by the exon 19/intron 19 “read-through” [Figure 1B–iii]. As a result of intron 19 retention, the truncated TOP2α/90 isoform does not harbor an active site tyrosine (Tyr805), which is present in the DNA gate domain [Figure 1B] and is required for wild-type TOP2α/170 to generate double-strand DNA breaks^[1–4]^. Finally, TOP2α/90 is also missing two characterized dimerization domains (DD) (i.e., 1053–1069 aa and 1121-1143 aa)^[41–44]^ and NLS 1454–1497^[46,47]^ present in wild-type TOP2α/170 [Figure 1B].

**Figure 3.**
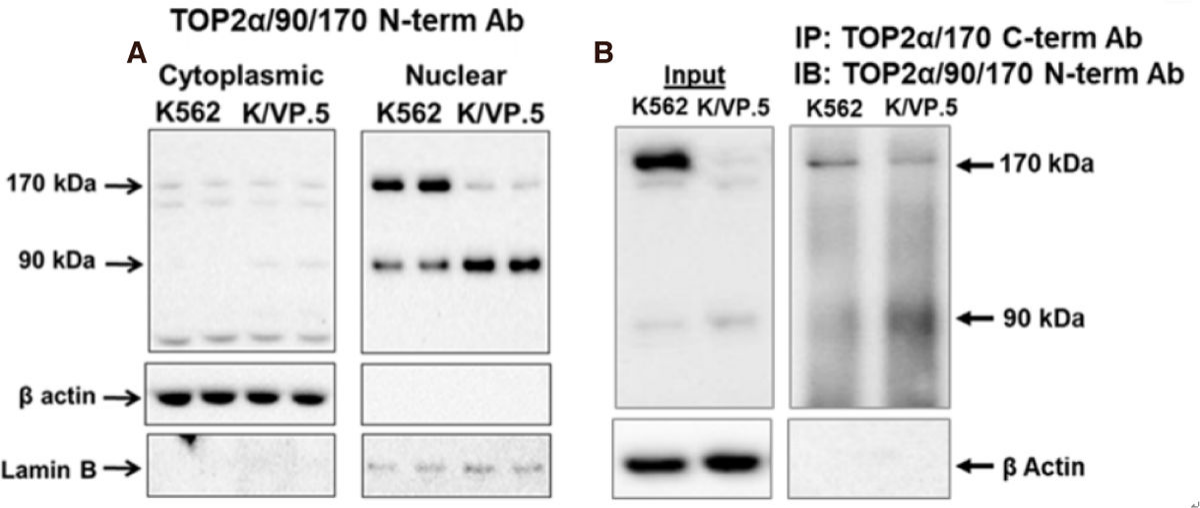
TOP2α/90 can be detected in both nuclear and cytoplasmic extracts and heterodimerizes with TOP2α/170. A: TOP2α immunoassay using K562 and K/VP.5 cytoplasmic and nuclear lysates^[36]^. Immunoblots were probed with TOP2α/90/170 and β-actin antibodies; B: immunoprecipitation experiments were performed using K562 and K/VP.5 whole cell lysates. The precipitated immune complexes were released in SDS-PAGE sample buffer, subjected to SDS-PAGE, and immunoblotted, using the indicated antibodies. Input immunoblots are also shown for each experiment and β-actin antibody loading controls. (A, B) Images are reproduced/adapted from Figures 2B and 3D, respectively, published originally in *Molecular Pharmacology*; Kanagasabai *et al.*^[36]^, 2018. TOP2α: topoisomerase IIα; SDS-PAGE: sodium dodecyl sulfate-polyacrylamide gel electrophoresis

It was hypothesized that, similar to the Top2α/160 truncated isoforms described above^[32–34]^, TOP2α/90 would be predominantly located in the cytoplasm since this isoform does not contain NLS 1454–1497 [Figure 1B]. Surprisingly, however, immunoassays using fractionated cytoplasmic and nuclear extracts [Figure 3A] and immunofluorescence experiments (not shown) demonstrated that TOP2α/90 was predominantly detected in the nucleus of K562 and K/VP.5 cells^[36]^. Currently, it is not known how TOP2α/90 is transported into the nucleus; a plausible speculation is that TOP2α/90 enters nuclei by a “piggy-back” mechanism^[58]^ (e.g., heterodimerization) with TOP2α/170, since the full-length isoform harbors functional NLS. In addition, TOP2α/90 may contain operative NLS sites. Mirski *et al.*^[47]^ found three bipartite NLS sequences in the first 743 TOP2α aa but these were not functional. A short non-classical IK-NLS motif^[58]^ (KVSKNK) in TOP2α/90 is currently under study for functionality.

Although TOP2α/90 does not harbor the DD essential for TOP2a/170:TOP2a/170 homodimerization [Figure 1B]^[41–45]^, co-immunoprecipitation experiments demonstrated that endogenous TOP2α/90 and TOP2α/170 proteins form heterodimers in both K562 and K/VP.5 cells [Figure 3B]^[36]^. While these results were unexpected, several studies have shown that human N-terminal TOP2α fragments, which encompass just the ATPase domain (i.e., aa 1–435), dimerize *in vitro* under the appropriate conditions^[59–61]^. Importantly, Bjergbaek *et al.*^[45]^ established that, if the C-terminal primary DD present in TOP2α/170 were deleted, dimerization could still occur in the presence of DNA and an ATP analog.

**Figure 4.**
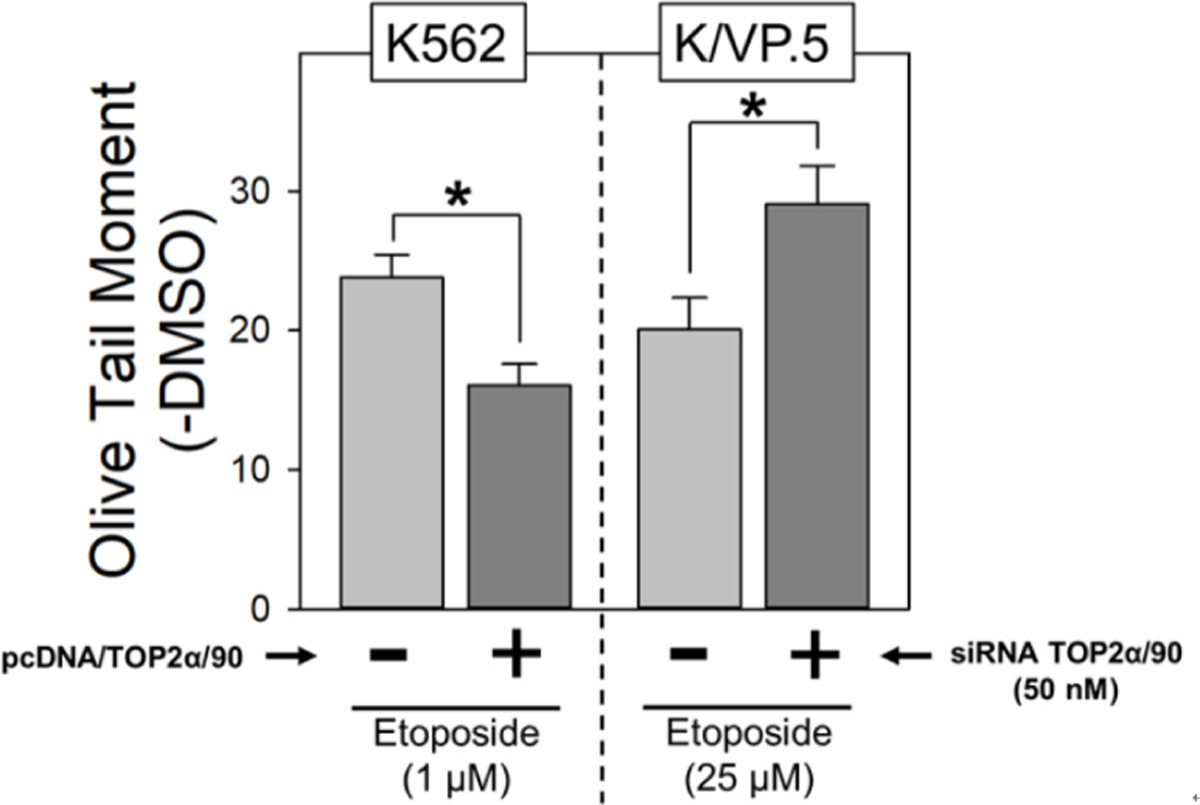
TOP2α/90 exhibits dominant-negative properties. A: etoposide (1 µM)-induced DNA damage in empty vector and pcDNA/TOP2α/90-transfected K562 cells was determined by neutral comet assays (assessing DNA double-strand breaks) after a 1-h incubation and subtraction of DMSO vehicle controls. The results shown are the mean ± SEM for three experiments run on separate days. *P < 0.025, comparing pcDNA/TOP2α/90-transfected to empty vector-transfected K562 cells; B: etoposide (25 µM)-induced DNA damage in negative control or TOP2α/90-specific Silencer Select Custom Designed TOP2α/90 siRNAs (50 nM) transfected K/VP.5 cells was determined as above. The results shown are the mean ± SEM for five to six experiments run on separate days. *P < 0.025, comparing TOP2α/90 siRNA-transfected to negative control siRNA transfected K/VP.5 cells. For all experimental conditions in each experiment, greater than 100 cells were evaluated by OpenComet software. (A, B) Images are adapted from Figures 4B (left) and Figure 5B (right) respectively, originally published in *Molecular Pharmacology*; Kanagasabai *et al.*^[36]^, 2018. TOP2α: topoisomerase IIα; pcDNA: plasmid cloning DNA; DMSO: dimethyl sulfoxide; siRNA: small interfering RNA

Given that TOP2α/90 lacks the active site tyrosine residue (Tyr805) required to form TOP2α/170-DNA covalent complexes [Figure 1B–iii], and is capable of heterodimerization with TOP2α/170 [Figure 3B], it was posited that this isoform may be dominant-negative relative to drug-induced DNA damage and cytotoxicity. Consistent with this hypothesis, forced overexpression of TOP2α/90 in K562 cells (which express low levels of Top2α/90) decreased etoposide-induced DNA damage and cytotoxicity in K562 cells [Figure 4]^[35,36]^. Conversely, etoposide-induced DNA strand breaks were increased in K/VP.5 cells subsequent to siRNA knockdown of elevated levels of TOP2α/90 [Figure 4]^[35,36]^.

Initial qPCR evaluation of paired AML patient samples (pre-treatment and relapse) indicated an increase in the ratio of expression of TOP2α/90 mRNA compared to TOP2α/170 after relapse^[36]^. In addition, the ratio of TOP2α/90 to TOP2α/170 protein was increased after treatment relapse^[36]^. To date, in four of six AML patients, there was a statistically significant increase in the TOP2α/90 to TOP2α/170 ratio after relapse (unpublished data). These results suggest a role for TOP2α/90 in resistance/relapse in patients and may lead to forward development of TOP2α/90 as a biomarker for development of drug resistance.

Overall, the studies described above strongly suggest that TOP2α/90:TOP2α/170 heterodimers produce dominant-negative effects by reducing the number of TOP2α/170-DNA covalent cleavage complexes that can be “trapped” by etoposide treatment. In turn, drug-induced DNA damage and cytotoxic action of etoposide are decreased. Therefore, we conclude that enhanced expression of TOP2α/90 in K/VP.5 cells is a determinant of chemoresistance through a dominant-negative effect related to heterodimerization with TOP2α/170. Given that TOP2α/90 mRNA is expressed in normal human tissues^[36]^, the formation of TOP2α/90:TOP2α/170 heterodimers may also play a role to protect against xenobiotics targeting TOP2α/170 or to “fine tune” levels of cleavage complexes.

Although this review focuses on variant pre-mRNA TOP2α/170 splicing in drug resistance, drugs that target type II topoisomerases also impact the 180 kDa isoform TOP2β/180, a separate gene product and not a splice variant^[62]^. Unlike TOP2α/170, TOP2β/180 levels are maintained throughout the cell cycle^[62]^. This isoform is important for transcriptional control and may play a role in drug-induced malignancies^[63,64]^. It is interesting to note that HL60/MX2 cells with intron 33 retention in TOP2α/170 have completely lost expression of TOP2/β protein^[32]^. In addition, K/VP.5 cells with intron 19 retention in TOP2α/170 do not seem to have similar alternative RNA processing of TOP2β based on qPCR evaluations across exon-exon junctions^[35]^. The paucity of information regarding potential splicing alterations in TOP2β in acquired drug resistance is a gap in knowledge which affords an opportunity for future investigations.

## CONCLUSION

Previous reports^[32–34]^, in conjunction with our newer studies^[35,36]^, support the conclusion that alternative TOP2α RNA processing is a determinant of acquired drug resistance and suggests that C-terminal truncated TOP2α isoforms may have additional biologic functions. Therefore, future studies are warranted to characterize the mechanisms by which alternative spliced TOP2α mRNAs are generated with the hope that these studies will lead to new strategies to circumvent acquired drug resistance. Further investigations may also lead to the development of tumor cell/biopsy evaluation of TOP2α isoforms as biomarkers for drug resistance, prognosis, and/or guide TOP2α-targeted therapies.
